# A comparison of transmission characteristics of *Salmonella enterica *serovar Enteritidis between pair-housed and group-housed laying hens

**DOI:** 10.1186/1297-9716-42-40

**Published:** 2011-02-23

**Authors:** Ekelijn Thomas, Annemarie Bouma, Don Klinkenberg

**Affiliations:** 1 Veterinary Public Health Division, Institute for Risk Assessment Sciences, Utrecht University, Yalelaan 2, 3584 CM Utrecht, The Netherlands; 2 Department of Farm Animal Health, Faculty of Veterinary Medicine, Utrecht University, Yalelaan 7, 3584 CL Utrecht, The Netherlands

## Abstract

Human cases of bacterial gastro-enteritis are often caused by the consumption of eggs contaminated with *Salmonella *species, mainly *Salmonella enterica *serovar Enteriditis (*Salmonella *Enteritidis). To reduce human exposure, in several countries worldwide surveillance programmes are implemented to detect colonized layer flocks. The sampling schemes are based on the within-flock prevalence, and, as this changes over time, knowledge of the within-flock dynamics of *Salmonella *Enteritidis is required. Transmission of *Salmonella *Enteritidis has been quantified in pairs of layers, but the question is whether the dynamics in pairs is comparable to transmission in large groups, which are more representative for commercial layer flocks. The aim of this study was to compare results of transmission experiments between pairs and groups of laying hens. Experimental groups of either 2 or 200 hens were housed at similar densities, and 1 or 4 hens were inoculated with *Salmonella *Enteritidis, respectively. Excretion was monitored by regularly testing of fecal samples for the presence of *Salmonella *Enteritidis. Using mathematical modeling, the group experiments were simulated with transmission parameter estimates from the pairwise experiments. Transmission of the bacteria did not differ significantly between pairs or groups. This finding suggests that the transmission parameter estimates from small-scale experiments might be extrapolated to the field situation.

## Introduction

Worldwide, many human cases of bacterial gastro-enteritis are caused by the consumption of eggs contaminated with *Salmonella *spp., mainly *Salmonella enterica *serovar Enteriditis (*Salmonella *Enteritidis) [1,2]. Reduction of human exposure is an important task for public health organizations and producers of poultry products. Consequently, the European Union (EU) has implemented a surveillance programme [3] to detect laying hen flocks colonized with *Salmonella *Enteritidis with the aim to reduce the number of contaminated eggs placed on the market.

The efficacy of a surveillance programme depends, amongst others, on sample size and frequency, which in turn depend on the within-flock prevalence. In surveillance programmes, sample size is often based on a fixed, static prevalence. The prevalence of *Salmonella *Enteritidis infected birds is, however, not a static characteristic of the flock, but changes over time since introduction of the bacteria into the flock. Therefore, surveillance programmes could be optimized if knowledge on time to reach a certain prevalence is included.

In general, the dynamics of a *Salmonella *Enteritidis infection in the flock depend on the susceptibility of hens for colonization, the number of *Salmonella *shed by colonized hens into the environment (e.g. litter), and the contact structure between colonized and susceptible hens. Parameters often used in studies about the dynamics are the transmission rate parameter *β*, defined as the mean number of secondary cases caused by one infected individual per unit of time, and the reproduction ratio *R*_0_, defined as the mean number of secondary cases caused by one infected individual in a susceptible population [4,5].

Quantification of these parameters can be done experimentally, which has the advantage over a field study that an infection chain can be deliberately started. This makes an experiment easier to carry out than field trials in which the moment of introduction of the bacteria is unknown. In a previous study we estimated these parameters *β *and R_0 _for *Salmonella *Enteritidis in laying hens housed pair-wise in cages [6]. The advantage of this design is that it is known which infectious animal infects which contact-exposed hen, and when this occurs. This creates the possibility to study the association between transmissibility and individual characteristics, such as shedding patterns [6], and also with the development of an immune response (IgY) in eggs, as eggs could be linked to individual chickens [7].

These results could be used to evaluate a surveillance programme based on egg sampling [8] and detection of IgY. For this evaluation, the results from the pairs should be extrapolated to large populations of laying hens as in large groups territories or sub-groups might be formed, possibly influencing the transmission rate or *R*_0 _[9-11]. The interaction between birds housed pair-wise is rather artificial, and may differ substantially from the interactions between animals in larger group. More specifically, it is important that the observed transmission rate from the pairs is correctly translated to groups.

The mathematical models used for analysis of this kind of data assume random mixing and frequency-dependent transmission [12], the validity of which was shown for pseudorabies virus in groups of 10 and 40 pigs [13]. Frequency-dependent transmission implies that the rate at which an infectious individual makes contacts is equal for all population sizes, but that in larger populations the contacts are distributed over more individuals. This assumption, however, allows two different interpretations. If a pathogen is transmitted by direct animal-to-animal contacts in a group of size *n*, the contact rate *β *of an infected animal is distributed over *n *- 1 animals, each animal receiving *β*/(*n *- 1) contacts. If a pathogen is transmitted indirectly, e.g. through excretions into the environment, an infected animal makes also contact with itself. This means that the contact rate *β *is distributed over *n *animals, each animal receiving *β/n *contacts. This may be a subtle difference when comparing transmission in groups of 10 and 40 pigs, but in the case of pairs it is a difference of a factor two. Thus, translation from pairs to groups requires special attention.

Because *Salmonella *Enteritidis is an environmentally transmitted bacterium, we used the second model, in which infected animals do have self-contacts [6,8]. In the current paper we test the validity of this assumption by comparing simulated outbreaks with outbreaks in three experimental groups of 200 laying hens. To study hypotheses explaining differences between experiment and simulation, some adjustments to the simulation model were considered in a scenario analysis.

## Materials and methods

### Animals

In total 780 laying hen hens of Lohmann brown classic (LB, *n *= 290) and white Lohmann selected leghorn classic (LSL, *n *= 490) breeds were purchased as 17-week-olds from two commercial grower poultry farms in The Netherlands. The hens had not been vaccinated against *Salmonella*. The farms were tested serologically for the presence of *Salmonella *antibodies, each on 12 randomly selected birds, and were found to be unsuspected of previous *Salmonella *infection before the purchase of the experimental laying hens. After a period of four days of acclimatization on the experimental facility, cloacal samples were collected from 270 randomly selected chickens on two consecutive days. These samples were subsequently cultured and tested negative for the presence of *Salmonella*.

All hens were individually tagged. The hens were provided with a commercial laying hen ration (Novex leg HP, De Heus B.V, Ede, The Netherlands) without any antibiotics, and were kept at 15 lux, with 14 h lighting. Each batch of feed was pelleted at 76°C, treated with 2 kg/1 000 kg formaldehyde (Sal CURB Dry, Kemin AgriFoods, Herentals, Belgium) and tested for presence of *Salmonella *according to ISO protocol ISO 6579:2002 [14]. Feed and tap water were available *ad libitum*.

The experiments were conducted at the Farm Animal Health Department, Faculty of Veterinary Medicine, Utrecht University and approved by the Animal Welfare Committee of Utrecht University under number 2008.III.06.058.

### Inoculum

The strain used in this experiment was a nalidixic acid and novobiocin resistant *Salmonella *Enteritidis phage type 4 (*Salmonella *Enteritidis PT4), courtesy of Paul Barrow [15,16]. The strain was taken from the -80°C freezer and transferred serially thrice in buffered peptone water (BPW) (Biokar Diagnostics, Pantin Cedex, France) and used for the inoculation at a dose of 1 * 10^9 ^colony forming units (CFU) for all experiments.

### Experimental design

Three experiments (1-3) were conducted with groups of 200 hens, and two experiments (4, 5) were conducted with 30 pairs of hens (the latter were described previously in Thomas et al. [6]). All experiments started when hens were 20 weeks old, the onset of lay. In each experiment the infection chain was started by inoculation of 4 birds in the groups of 200 (exp 1-3) or one bird per pair (exp 4, 5). In the groups of 200, in contrast to what is assumed to occur in the field that an infection starts with one infected bird, the infection was started with four seeders. The transmission model used to analyze the experiments can handle any initial situation. We chose four seeders as a compromise between "guaranteeing" that a major outbreak is observed and staying close to what is thought to be a natural way of the start of colonization of a flock [17].

#### Experiments with 200 laying hens

##### Experiment 1

This experiment was carried out with 200 Lohmann brown classic. The hens were housed in a saw dust litter system divided into a littered area for foraging, feeding, and drinking, and a raised nesting area above a droppings pit (1/4 of the total surface), according to Council Directive 1999/74/EC [18]. The density was 9 hens/m^2 ^litter area, equivalent to 6.9 hens/m^2 ^total area.

On day -1 (D-1) of the experiment, four randomly selected hens were removed and put in a separate crate. These hens were inoculated intra-esophagically with 1 mL (1 * 10^9 ^CFU) of the inoculum *Salmonella *Enteritidis PT4. One day later, all inoculated hens were placed back into the group (day 0). Thus at the start of the experiment, the group consisted of four inoculated and 196 contact-exposed (in-contact) birds. The experiment ended at D39.

Cloacal swabs were collected from 50 randomly selected birds on days 1-9, (50 randomly selected hens were sampled on each of these days), 11-16, 18, 19, 26, 33 and 39. Following Commission Regulation 1168/2006 procedure [3], two pairs of boot swabs were collected weekly after inoculation, and once before inoculation. Thirty eggs were collected on days -1, 7-22, 25.

##### Experiment 2

This experiment was carried out with white Lohmann selected leghorn classic, identically to experiment 1 with some small adjustments. Although droppings pits are common in commercial laying hen farms in barn housing, the pit was covered to allow physical contact between birds and excreta. At D13, four seeders were removed from the experiment. Four previously contact-exposed were inoculated and introduced at D14. The experiment ended at D42.

Cloacal swabs were collected from all inoculated birds on D0. Cloacal swabs were collected from 50 randomly selected birds on days 1-9, 11-13, 16-22 and 42.

Two pairs of boot swabs were collected weekly after inoculation, and once before inoculation. Thirty eggs were collected on days -1-39.

##### Experiment 3

Experiment 3 was carried out with white Lohmann selected leghorn classic, as described for experiments 1 and 2. The droppings pit was covered. The experiment ended at D54.

Cloacal swabs were collected from all birds inoculated on D0. Cloacal swabs were collected from 50 randomly selected hens on days 1-9, 11-15, 18-22, 39, 42, 46, 50 and 54 - with the exception of days 25, 28, 32 and 35, on which 10 random cloacal swabs were taken. Two times per week 10 randomly selected hens were euthanized after cloacal swab sampling. After having opened the abdomen, an additional caecal swab was taken. The floor area was diminished with 2 m^2 ^weekly, so as to keep the density at 9 hens/m^2 ^litter area. Two pairs of boot swabs were collected weekly after inoculation, and once before inoculation. Thirty eggs were collected on days -1-2, 4-10, 12-25, 28-54.

#### Experiments with 2 laying hens

##### Experiments 4 and 5

The experiments with two laying hens per group have been described by Thomas et al. [6]. Briefly: two replicates of 90 hens were allocated to 2 × 3 groups of 30 birds each and each group was housed in a separate room. In each room 10 pairs of hens were put in a saw dust litter cage at a density of 8 hens/m^2^, and 10 hens were housed individually in cages in between as sentinels. Each pair consisted of one white and one brown hen, producing white and brown eggs, respectively. On day -1 of the experiment, one randomly selected hen from each pair was removed and kept separate alone in a cage in the same room (five brown and five white per room). These hens were inoculated and reintroduced as described for Experiment 1. The sentinels were not inoculated. The experiments ended at D26 (experiment 4); at D32 (experiment 5, room 1); at D39 (experiment 5, room 2); and at D45 (experiment 5, room 3).

On day 1, sampling of cloacal swabs from both inoculated, contact-exposed and sentinel hens was started and continued on days 2-9, 11-14, 16 and 18. In experiment 5, cloacal swabs were collected from all inoculated hens on D0. Eggs were collected at days -1, 9, 12-16, 19-21, and 26 in experiment 4 and on a daily basis from day 7 until the end of the experiment 5.

Cloacal swabs were put into tubes containing 10 mL sterile buffered peptone water (BPW, Biokar Diagnostics, Pantin Cedex, France) at room temperature and were transported for bacteriological examination that same day. Eggs were stored at 4°C. Boot swabs were put into sterile plastic bags and were transported for bacteriological examination.

At the end of the experiments, all hens were euthanized by cervical dislocation after cloacal swab sampling (except for experiment 4). With the exception of experiments 1 and 4, after having opened the abdomen, an additional caecal swab was taken, which was treated as described for cloacal swabs.

### Bacteriology

To detect *Salmonella *shedding before inoculation, cloacal swabs were cultured following the procedures recommended by ISO 6579:2002 [14].

To detect *Salmonella *shedding after inoculation, cloacal en caecal swabs were enriched in BPW (18 ± 2 h, 37°C) and cultured on Rappaport-Vassiliadis medium, semi-solid modification (MSRV) with 0.02 g/L novobiocin (CM910, Oxoid, Basingstoke, UK) (24 ± 3 h, 42°C ± 1°C). Negative cultures were reincubated for a further 24 h ± 3 h. Regular confirmation of positive colonies was performed by biochemical confirmation on trypton soya iron and urea agar, and lysine Decarboxylase medium (Biotrading, Mijdrecht, The Netherlands) and serum agglutination (Pro-lab diagnostics, Neston, UK). *Salmonella *Enteritidis PT4 and an in-house *Escherichia *coli K12 strain were used as positive and negative controls respectively throughout the bacteriological procedures.

Boot swabs were cultured per pair according to ISO 6579:2002 [14].

### Immunology

Preparation of egg samples as well as the method of suspension array analysis was described earlier [7]. In short: suspension array analysis (Cell lab Quanta™ SC, by Beckman Coulter B.V., Mijdrecht, The Netherlands) was used for detection of chicken egg yolk antibodies against lipopolysaccharide (LPS) of *Salmonella *Enteritidis using polystyrene beads coated with LPS of *Salmonella *Enteritidis (RnA BV, Utrecht, The Netherlands). Samples were diluted in PBS buffer, and filtrated. Beads were added to egg yolk allowing anti-*Salmonella *antibodies to bind to the antigen. A second and third incubation step involved biotinylated donkey anti-chicken and streptavidin label, respectively. Between 15' incubation steps were washing steps. After the last incubation step, the beads were resuspended in assay buffer, and the fluorescent intensity of the label on each sample was then measured with suspension array analysis and expressed in median fluorescence intensity (MFI). The label's fluorescent intensity is proportional to the amount of anti-*Salmonella *antibodies on the bead. A hen was considered to have been colonized as she had at least one *Salmonella *Enteritidis culture positive cloacal swab sample during the observational period.

For this test, estimates of specificity, sensitivity on hen level and on sample level, and the sensitivity as a function of time since excretion are available [7].

### Statistical analysis: modeling *Salmonella *Enteritidis transmission

The aim of this study was to assess whether transmission parameters estimated from pairwise experiments could be extrapolated to larger groups. This was done by comparing the results of the group experiments to the outcome of computer simulations of the course of infection in these experiments, using the mathematical model and the transmission parameters estimated from the pairwise experiments. Simulations were carried out in three stages:

(1) simulation of the transmission process, resulting in times of infection for each hen

(2) simulation of the egg sampling and testing process, resulting in numbers of positive eggs on each sampling day

(3) simulation of the cloacal swab sampling and testing process, resulting in numbers of positive cultures on each sampling day.

Five sets of simulations (scenarios) were done for each of the three experiments:

A. Baseline simulation, with all parameters estimated from the pairwise experiments

B. As A, but with bacterial culture parameters estimated from the group experiments, for each experiment separately. This means that the parameter estimates from the pairwise experiments were used for simulating the transmission and the egg testing, but that the culture sensitivity was estimated from the group experiments (*cf*. section "*Model 3: bacteriological sampling and testing*")

C1. As B, but with a lower transmission rate (75% of original)

C2. As B, but with a higher transmission rate (150% of original)

C3. As B, but with a lower sensitivity of the egg test (75% of original) and a delayed immunological response (5 days later).

The mean course (and 95% interval) of 1 000 simulations was plotted and compared to the outcome of the experiments. In addition, some summary statistics were calculated and compared between simulations and experiments:

- the total number of culture-positive samples, *C_total_*

- the number of culture-positive samples on the last sampling day, *C_end_*

- the total number of egg-positive samples, *E_total_*

- the number of egg-positive samples on the last sampling day, *E_end_*.

#### Model 1: transmission

The transmission process was simulated in time steps of 0.01 day. At time *t *= -1, it was started with four infected hens, with infection times *t^i^*_(1) _= *t^i^*_(2) _= *t^i^*_(3) _= *t^i^*_(4) _= -1. At each next time point *t *the following two steps were taken:

1. The total force of infection Λ(*t*) was calculated from the *I_t _*infected hens, which had been infected at times *t^i^*_(1)_, *t^i^*_(2)_,..., *t^i^*_(*It*)_:

$$\Lambda \left(t\right)=\sum_{j=1}^{I_{t}}\beta_{\left(j\right)}(t-t^{i}{}_{\left(j\right)}).$$

**Figure 1:**
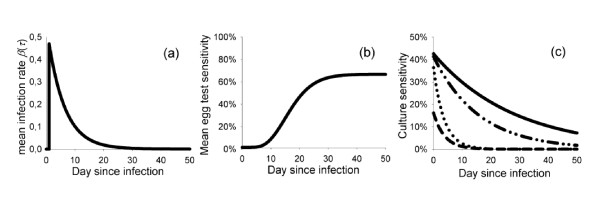
**Graphical description of the simulation model.** (a) Model 1 (transmission): the rate at which hens in a laying flock are colonized as a result from *S*. Enteritidis excreted by a single hen (*i*). Model for a hen with mean infectivity level (*X*) of 6.8, based on the pairwise experiments; (b) Model 2 (immunological sampling and testing): sensitivity of egg yolk immunology as a function of time since colonization, based on the pairwise experiments; (c) Model 3 (bacteriological sampling and testing): sensitivity of cloacal swab culture as a function of time since colonization, based on the pairwise experiments (___), group-experiment 3 (_ .. _ ..), group-experiment 1 (....), and group-experiment 2 (_ _ _).

Thus, the force of infection is the sum of each individual infectiousness *β *_(*j*)_(τ) of the *j*^th ^infected hen, at time τ since that hen was infected itself (Figure 1a) [6]:

$$\begin{array}{ll} \beta_{\left(j\right)}\left(\tau \right)=0 & \tau <1\\ \beta_{\left(j\right)}\left(\tau \right)=\left(b_{0}+b_{1}X_{\left(j\right)}\right)\exp\left(-\gamma \tau \right) & \tau \geq 1. \end{array}$$

The individual infectiousness consists of a one-day latent period, followed by a high infectivity level that is immediately followed by a slowly declining infectivity level. The initial level differed for each hen through the random variable *X*_(*j*)_, which was normally distributed with parameters *μ_X _*and σ*_X_*. In scenarios C1 and C2, *β *_(*j*)_(τ) was multiplied by 0.75 and 1.5, respectively.

2. The number of new cases at time *t *is sampled from a binomial distribution with *n *= *S_t _*and *p *= 0.01 αΛ(*t*)/(*S_t _*+ *I_t_*). Here, *S_t _*is the number of susceptible hens, *α *is a correction factor for the number of birds per unit of area (*α *= 1 for the pairwise experiments, *α *= 6.9/8 for the group experiments), and 0.01 is the time step size. The new cases *j*+1, *j*+2, etc. are attributed infection time *t*.

3. At the appropriate time points in the simulations of experiment 3, hens were randomly removed.

In total, 1 000 simulations were carried out per scenario. For each simulation, a unique parameter set was sampled using the pairwise experimental data, the likelihood function in Thomas et al. [6], and the maximum log-likelihood *L*_max_. Samples were taken from the 95% likelihood ratio confidence set of all parameters (Table 1), by the following algorithm:

**Table 1:** Parameter values for the simulations; (a) maximum likelihood estimates with 95% confidence intervals (models 1 and 2, Thomas et al. [6, 7, respectively]); (b) posterior medians and 95% credible intervals (model 3).

**Model 1**		**Model 3**		
				
*b*_0_	0.13 [0; 0.40]	*Data source*		
*b*_1_	0.051 [0.014; 0.094]	Pairs	*Se*_max_	0.43 [0.32; 0.54]
*γ_β_*	0.17 [0.09; 0.25]		*γ_Se_*	0.035 [-0.0004; 0.070]
*μ_X_*	6.8 [5.8; 7.7]	Group-exp. 1*	*Se*_max_	0.36 [0.16; 0.83]
σ*_X_*	3.6 [3.0; 4.4]		*γ_Se_*	0.27 [0.12; 0.52]
		Group-exp. 2	*Se*_max_	0.16 [0.05; 0.53]
				
**Model 2**			*γ_Se_*	0.26 [0.03; 0.88]
Pr*_hen_*	0.67 [0.47; 1.0]	Group-exp. 3	*Se*_max_	0.41 [0.28; 0.60]
*μ_egg_*	17 [14; 27]		*γ_Se_*	0.06 [0.03; 0.11]
*ν_egg_*	6.9 [2.8; 14.1]			
Pr*_egg_*	0.98 [0.95; 0.99]			
Sp	0.99 [0.98; 0.99]			

* short for "group-experiment"

(a) sample the five parameters uniformly from their individual confidence intervals (Table 1) and calculate the log-likelihood *L*_sample_

(b) if 2(L_max _- L_sample_) < 3.84 (χ^2^_(1)_-distribution), then accept the sample, otherwise restart at (a).

Each simulation resulted in the infection times of all hens, which were used in the simulations of the sampling and testing process.

#### Model 2: immunological sampling and testing

The analysis of the egg test [7] has revealed that only a proportion Pr_hen _of all infected hens immunoconverted, as indicated with an antibody response detectable in eggs. If a hen did, the time between infection and immunoconversion was gamma-distributed with mean *μ*_egg _and shape parameter ν_egg_. After that time, the probability of a positive egg was Pr_egg_, whereas eggs laid before that time, and eggs by hens that did not immunoconvert, had a probability 1 - Sp to be positive. The parameter values are listed in Table 1. The mean sensitivity of egg yolk immunology as a function of time since colonization is depicted in Figure 1b.

The following steps were taken to simulate the number of immunopositive eggs on each sampling day:

1. For each infected hen it was sampled whether it had immunoconverted

2. For each immunoconverted hen, the day of immunoconversion was sampled

3. On each sampling day, the number of true-negative and true-positive hens was determined, a sample was taken (sample size as in the experiment), and the number of positive eggs was obtained by sampling the test result for each egg.

For each simulation, a different parameter set was used, which was obtained as described for model 1, by use of the data and likelihood function described in Thomas et al. [7].

#### Model 3: bacteriological sampling and testing

For the detection by bacteriological culture in all experiments, the following detection probability per sample was used, as a function of time since infection:

$$Se\left(\tau \right)=Se_{\max}\exp\left(-\gamma \tau \right).$$

For scenario A (baseline), the parameters *Se_max _*and γ were estimated from the pairwise experiments. For scenarios B and C, they were estimated from the three group experiments separately (Figure 1c). The estimation procedure is described below.

The specificity of the test was assumed to be 1.

The following steps were taken to simulate the number of culture-positive hens on each sampling day:

1. On each sampling day, a sample of hens was taken (sample size as in the experiment), and for each hen the probability of detection was determined

2. The number of positive hens was obtained by sampling the test result for each hen in the sample.

For each simulation a different set of parameters was used. The sets of parameters were samples from the posterior distribution of the parameters, obtained by Bayesian estimation with data from the pairwise experiments (scenario A), and from the group experiments (scenarios B, C1, C2, C3).

For estimation of the parameters of the bacterial culture model, all hens were considered to be independent. Culture data were available for the sampling days, with limited data for the group experiments because each day only part of the hens was sampled. Posteriors were obtained for the probability that a hen got infected during the experiment (*p_inf_*), the time that each hen *i *got infected (*t_inf, i_*), the initial sensitivity of the test (*Se_max_*), and the rate of the sensitivity decrease (γ). Prior distributions were uniform for all these parameters and variables: *p_inf _*~ U(0,1), *Se_max _*~ U(0,1), γ ~ U(-1,1), and all *t_inf, i _*were uniformly distributed between 0 and the time of the first positive sample or the end of the experiment, whichever came first. The analyses were carried out in WinBugs 1.4 [19]. After a burn-in of 1 000 iterations, every 10^th ^iteration was included to complete a set of 1 000 samples.

## Results

### Descriptive analyses

The inoculations were considered successful in all five experiments with at least one culture-positive result from the cloacal swabs per inoculated bird. Shedding had often started already at day 0 (the day after inoculation, as sampled in experiments 2, 3, 5).

**Figure 2:**
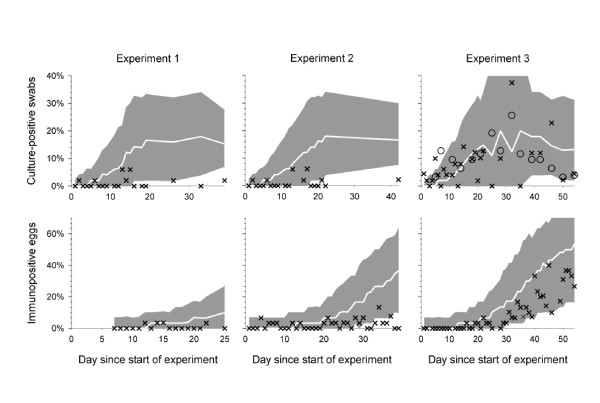
**Course of simulated *S. Enteritidis* outbreaks in groups of 200 laying hens: baseline simulation scenario A**. Top panels: % culture-positives with time; lower panels: % egg-positives with time. Simulations: the white line denotes the 50^th ^percentile of simulation results for every sampling day, within the 90% grey area. Experimental data: "X" denote cloacal swab culture-positives or egg-positives, respectively; "O" denote proportion positive cecal swab culture (experiment 3 only).

Figure 2 shows the proportions culture positive results at all sampling days, in the three group experiments. In experiments 1 and 2, with 4 seeders and 196 contact-exposed, in total 23 in experiment 1 and 10 hens in experiment 2 had at least one culture-positive result from the cloacal swabs during the study periods of 39 and 42 days, respectively. In experiment 3, 59 of 196 of contact-exposed birds tested positive within a study period of 54 days. In experiments 4 and 5 with one inoculated and one contact-exposed bird, 23 and 18 of 30 contact-exposed birds became culture-positive, respectively. The mean number of positive cultures per hen from experiments 4 and 5 did not differ significantly between replicates or between breeds [6].

At the end of the experiments, after euthanasia of all remaining hens, 2 out of 190 available caecal swabs were culture-positive (experiment 2), 6/49 (experiment 3) and 3/86 (experiment 5). In 54 hens of experiment 3, culture results of additional 10 caecal swabs per sampling, taken *during *the experiment, were positive (*cf*. section "*Experiments with 200 laying hens"*). For 53 of those positive caecal swabs, a parallel cloacal culture result from the same hen was available and 17/53 cloacal swabs were culture-positive, indicating a poor sensitivity of cloacal swab culturing. To allow for comparison of the observed caecal swabs results with the simulated cloacal swab results, and only for the purpose of showing the cloacal swab results in the same figure, the proportions culture-positive were multiplied by 17/53 (Figure 2).

The samples with caecal swabs showed a gradual increase in prevalence with time from 1/10 at D5 to 6/10 at D25, up to a peak of 8/10 at D32 and then declined gradually to 1/10 at D50 (Figure 2). Both pairs of boot swabs that were collected weekly in experiments 1-3 were culture-positive until the end of the respective study periods (except in experiment 2, in which only one boot sample was culture-positive on days 11 and 18).

In experiments 1-3, a random sample of 30 eggs was collected almost daily and the yolks were tested for antibodies against *Salmonella *Enteritidis. After acclimatization, all birds were found to be unsuspected of previous *Salmonella *infection by egg antibody detection on day -1. In experiment 3 only, a marked increase was observed of the proportion egg-positives in time, from 0.03 to 0.40 between about 4 and 8 weeks after introduction of 4 seeders (Figure 2). In experiments 4 and 5, at D26, 9/30 and 11/30 of contact-exposed hens had a positive egg antibody test.

### Statistical analysis: modeling *Salmonella *Enteritidis transmission

The pairwise experiments resulted in an estimated (median of posterior distribution) sensitivity of the cloacal swab culture of 43% shortly after colonization, which decreased to 15% after 30 days (Table 1; Figure 1c).

**Figure 3:**
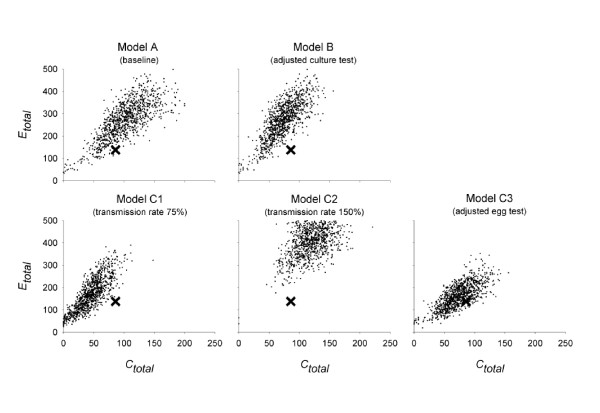
**Scenario analyses group-experiment 3: scatter plots of the sum of culture-positives (*C_total_*) and egg-positives (*E_total_*) for each of 1 000 simulations**. Five scenario's are depicted; A) baseline simulations based on parameter estimates derived from the pairwise experiments; B) as A), but with bacterial culture parameters estimated from the group experiments, for each experiment separately; C) as B), C1) but with 75% of the original transmission rate, C2) but with 150% of the original transmission rate, C3) but with 75% sensitivity of the egg test and a delayed immunological response of 5 days. "X" denote the results from experiment 3.

Figure 2 shows the mean course of the experimental outbreaks and the course of simulations with baseline scenario A. The simulated outbreak of experiments 1 and 2, but not of experiment 3, resulted in higher estimates of the prevalence of colonized birds based on bacterial cultures. In all three experiments, the simulated egg tests resulted in an immune response that arose too early compared to the data. All observed numbers of positive tests were consistently lower than in the simulations in experiments 1 and 2 (Table 2). For experiment 3, data and simulations did match. However, when looking at the scatterplot of *C_total _*against *E_total_*, the observed data were outside the simulated range (Figure 3).

**Table 2:** Summary statistics of the comparison between results from 1 000 simulations and group-experiments 1-3, expressed as the total number of culture-positive samples (*C*_*total*_) and egg-positive samples (*E*_*total*_), and the number of culture-positive samples (*C*_*end*_) and egg-positive samples on the last sampling day (*E*_*end*_).

Experiment		Experimental data	Simulation scenario's				
			**A**	**B**	**C1**	**C2**	**C3**
			
**1**	*C_total_*	25	18-129	4-55	2-36	16-82	4-55
	*C_end_*	0	17-58	0-9	0-10	0-4	0-9
	*E_total_*	4	8-37	8-37	7-30	12-57	4-20
	*E_end_*	0	0-8	0-8	0-6	1-13	0-4

**2**	*C_total_*	10	17-137	2-26	0-17	8-36	2-26
	*C_end_*	4	14-54	0-6	0-6	0-5	0-6
	*E_total_*	28	52-216	52-216	35-162	120-306	29-116
	*E_end_*	0	3-19	3-19	1-14	10-23	1-12

**3**	*C_total_*	86	34-170	27-123	2-86	69-171	27-123
	*C_end_*	2	1-14	0-7	0-7	0-6	0-7
	*E_total_*	138	98-424	98-424	49-323	264-538	61-268
	*E_end_*	8	5-23	5-23	1-19	12-26	3-18

**Figure 4:**
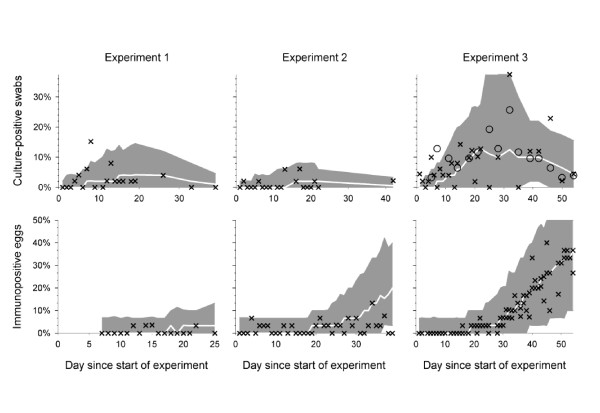
Course of simulated *S. Enteritidis* outbreaks in groups of 200 laying hens: simulation scenario C3 with lower sensitivities of cloacal swab test and egg test.

For the first alternative scenario (B), we first estimated the sensitivity parameters for bacterial culture from the data of the three group experiments, separately for each experiment. This resulted in lower initial and more rapidly decreasing sensitivity, more pronounced for experiments 1 and 2 than experiment 3 (Table 1; Figure 1c). Simulations with the alternative model improved the match of the bacteriological test results (Figure 4 top panels; Table 2). Because the adjustment of the culture model was based on experimental data, we kept this adjustment in place while exploring three other alternatives in an effort to explain the differences between simulations and data.

In the three alternatives C1-3, we decreased (C1) or increased (C2) the transmission rate, or adjusted the egg test characteristics (C3). Table 2 shows that a lower *Salmonella *Enteritidis transmission rate led to a lower number of positive eggs, moving towards the observed data. However, looking at *C*_*total *_and *E_total _*together, it appeared that changing the transmission rate did not bring data and simulations closer together (Figure 3). Only adjustment of the egg test characteristics could really improve the model based on statistics (Table 2), scatterplot (Figure 3), and the time course of the outbreak (Figure 4).

## Discussion

The aim of this study was to compare infection dynamics of *Salmonella *Enteritidis in pairs of laying hens with the dynamics in groups of 200 hens housed at similar densities, and to test whether the mathematical model used to analyze transmission in pairs was valid to be used for larger groups. There was no indication for violation of the random mixing assumption: the transmission process was not different in groups in comparison to pairs. This means that the transmission parameters, quantified in pairwise experiments, may be extrapolated to larger groups. Whether the results can be extrapolated to the field remains to be determined, as conditions in experiments and in the field are different in many ways.

This conclusion (no difference between transmission rates) was reached in spite of the fact that differences between immunoprevalence as well as prevalence based on culture were observed. In the first place, contact-colonized hens in group experiments 1 and 2 often had negative results of repeated cloacal swab samplings, below the simulated number that was expected based on the observations in the pairs. In addition, as compared to the simulations, the observed number of positive egg tests was too low (group experiments 1-2) and the immune response arose too late (group experiments 1-3). In scenario analysis, it appeared that changing the transmission rate to 75% or 150% of the original value did not at all bring data and simulations closer together (Figure 3), thus indicating that the transmission process was not different in groups with different sizes. Therefore, as a possible explanation for the observed differences, lower diagnostic sensitivity of bacterial and immunological sampling and testing was further explored.

The first indication of a sensitivity problem was observed in experiment 3, in which sensitivity of detection was more than tripled by additional caecal swab sampling and testing [20]. Then, a closer look at the cloacal swab data revealed that contact-colonized hens in group experiments 1 and 2, after an initial positive swab, often had negative results of repeated cloacal swab samplings: more negative results than expected from the observations in the pairs. Because of this, we adapted the culture model parameters by using the experimental data from each separate group experiment. In the scenario analysis, it appeared that this lower sensitivity of detection by culture could well explain the observed difference in prevalence. It should be noted that caecal swabbing during the trial was not chosen originally, because it can only be done post-mortem, resulting in a decrease of group size. By keeping the animal density at the same level, however, extrapolation of results from the pairs to the groups by means of the mathematical model remained possible.

Between simulations based on the pairs and results from the group experiments, immunoprevalence based on the egg-test differed as well. This can be explained by lower test sensitivity. Variation in test sensitivity was also seen in the pairs, where higher egg-test sensitivity was found in inoculated hens than in contact-colonized hens [7]. However, the sensitivity curve of the egg test could not be re-estimated for the groups from the available data, because eggs could not be related to the individual hens. If egg-test sensitivity in groups of hens is indeed lower, this might limit the use of egg immunology in *Salmonella *surveillance systems.

Prevalence based on cloacal swab culture appeared to differ between pairs and groups. The difficulties with testing based on bacterial culture for *Salmonella *Enteritidis may have occurred as a result of inoculation of 4/200 chickens instead of 50% as in the pairs, resulting in lower environmental contamination and shedding of a lower number of bacteria (below the detection limit) by contact-colonized birds in the groups. A different experimental design, inoculating 100/200 hens, would not have been a better choice though, because the transmission rate is not related to the number of seeders but only depends on the numbers of infectious and susceptible birds present. Indeed, the lower numbers of bacteria did not affect the chance of excretion nor did it decrease the transmission rate, which is consistent with the findings of Velkers et al. for *E. acervulina *in broilers [21].

We did not compare the results with observations from the field, because it is difficult to perform field studies, not only because of practical reasons, but also because many flocks in The Netherlands are vaccinated against *Salmonella *Enteritidis. However, we are convinced that our transmission model, quantified from pairwise experiments, can well be extrapolated to larger groups and is therefore the best model available for commercial flocks so far.

## Competing interests

The authors declare that they have no competing interests.

## Authors' contributions

All authors participated in the design of the study. MET carried out the experiments and drafted the manuscript. AB carried out the experiments. DK carried out the statistical analysis. All authors contributed to the manuscript, and read and approved the final version.
